# Development of perivascular astrocyte processes

**DOI:** 10.3389/fnins.2025.1585340

**Published:** 2025-07-04

**Authors:** Martine Cohen-Salmon, Naomie Guille, Anne-Cécile Boulay

**Affiliations:** Center for Interdisciplinary Research in Biology (CIRB), Collège de France, CNRS, INSERM, Université PSL, Paris, France

**Keywords:** astrocytes, blood vessels, perivascular astrocyte processes, endfeet, gliovascular unit, neurovascular unit, brain, brain interface

## Abstract

Astrocytes are key glial cells in the brain that form specialized contacts with the vascular system. Together, these interactions constitute the gliovascular unit (GVU), which is an interface between the brain and the blood crucial for the maintenance of the structure and functions of the brain. The development of the GVU is a complex process involving multiple steps and intricate interactions among astrocytes, neural cells, and vascular components. In this review, we aim to summarize the current understanding of the development of the astrocyte–vascular interface and to explore how early developmental alterations in this system may contribute to brain dysfunction.

## 1 Introduction

The development of the brain’s vascular interface is a tightly orchestrated process that underpins many aspects of central nervous system (CNS) function. At the heart of this interface are perivascular astrocyte processes (PvAPs)—specialized extensions of astrocytes that physically and functionally connect neural tissue to the cerebrovascular system. These structures are central to the formation and maturation of the gliovascular unit (GVU), contributing to key processes such as blood–brain barrier (BBB) formation, neurovascular coupling, and metabolic support. Despite their critical roles, how PvAPs develop remains incompletely understood. In this review, we synthesize current knowledge on how PvAPs emerge, organize, and interact with other cellular components of the brain vasculature during development, and we examine how early alterations in these processes may affect brain functions.

Astrocytes are highly ramified glial cells, best known for their star-like morphology—a feature first described in the pioneering work of Santiago Ramón y Cajal and Camillo Golgi. This characteristic shape primarily reflects the organization of their intermediate filaments cytoskeleton, which is composed of glial fibrillary acidic protein (GFAP) fibers. More recently, imaging techniques using fluorescent dyes filling the cell cytoplasm have enabled visualization not only of astrocytes’ main branches but also of their full three-dimensional structure, including multiple orders of fine processes that form a bushy architecture. Under normal conditions, each of these astrocytic “bushes” defines a distinct territory (Bushong et al., 2002), within which thousands of synapses are embedded. Astrocytic contacts with synapses are essential for the regulation of synaptic functions. The concept of the “tripartite synapse” was introduced to describe the bidirectional communication between neurons and astrocytes (Araque et al., 1999). In addition to their interactions with neurons, astrocytes also contact blood vessels (BV), with specialized structures historically known as the endfoot. At this interface, astrocytes play a crucial role in regulating BBB, which controls the selective passage of nutrients while preventing the entry of potentially harmful substances and peripheral immune cells. Astrocytes also participate in regulating neurovascular coupling by sensing neuronal activity and modulating BV diameter to match local metabolic demands. Furthermore, they help maintain homeostasis in the brain’s extracellular environment by regulating ion and neurotransmitter concentrations—processes essential for normal neuronal function. Additionally, astrocytes contribute to brain fluid drainage by organizing the flow of cerebrospinal (CSF) and interstitial (ISF) fluids. These diverse and vital roles depend on the expression of a specific molecular repertoire by astrocytes at the vascular interface and the release of signaling molecules that influence the properties and functions of vascular cells.

## 2 Structural organization of perivascular astrocyte processes (PvAPs)

Images of astrocytes filled with fluorescent dyes show not only processes terminated by a unique and homogenous “foot’ but also less structured perivascular contacts with BVs (Bindocci et al., 2017; Hösli et al., 2022). Therefore, the term endfoot is probably not encompassing the diversity of this compartment. It was also named “astrocyte-vessel area” (Hösli et al., 2022), or perivascular astroglial sheath (Mathiisen et al., 2010). More recently the term perivascular astrocyte processes (PvAPs) was used by analogy with the perisynaptic astrocyte processes (PAP; Avila-Gutierrez et al., 2024; Skauli et al., 2024), which better represents both structural and functional aspects of this astrocytic compartment. Therefore, we will use this term here. In hippocampal astrocytes, PvAPs account for approximately 3.5% of the total astrocytic volume (Bindocci et al., 2017). Electron microscopy (EM) studies of the hippocampus have shown that astrocyte perivascular coverage by PvAPs is nearly complete (Mathiisen et al., 2010). However, the concept of a continuous and complete astrocytic perivascular coverage is now being challenged. Direct microglial-vascular contacts have recently been observed in the adult mouse frontal cortex occupying about 4% of the capillary surface (Mondo et al., 2020; Morris et al., 2024). Contacts between the vasculature and oligodendrocytes or their precursors (OPC) have also been reported (Su et al., 2023). A recent study showed that discontinuities in astrocyte arteriolar coverage could be occupied by glutamatergic presynapses connected to vascular smooth muscle cells (VSMC) and implicated in the regulation of the neurovascular coupling (Zhang et al., 2024). Finally, serotoninergic synapses abutting the basal lamina (BL) have been observed in the rat cerebral cortex and hippocampus (Cohen et al., 1995). In the cortex, midbrain, thalamus, and hypothalamus, all astrocytes contact BVs and in the hippocampus, only a small proportion (2.6%) of astrocytes lack vascular contacts (Hösli et al., 2022). Observations also suggest that cortical and hippocampal astrocytes typically contact an average of three capillary segments (Bindocci et al., 2017; Hösli et al., 2022). This number correlates with capillary density (Hösli et al., 2022).

**Figure 1:**
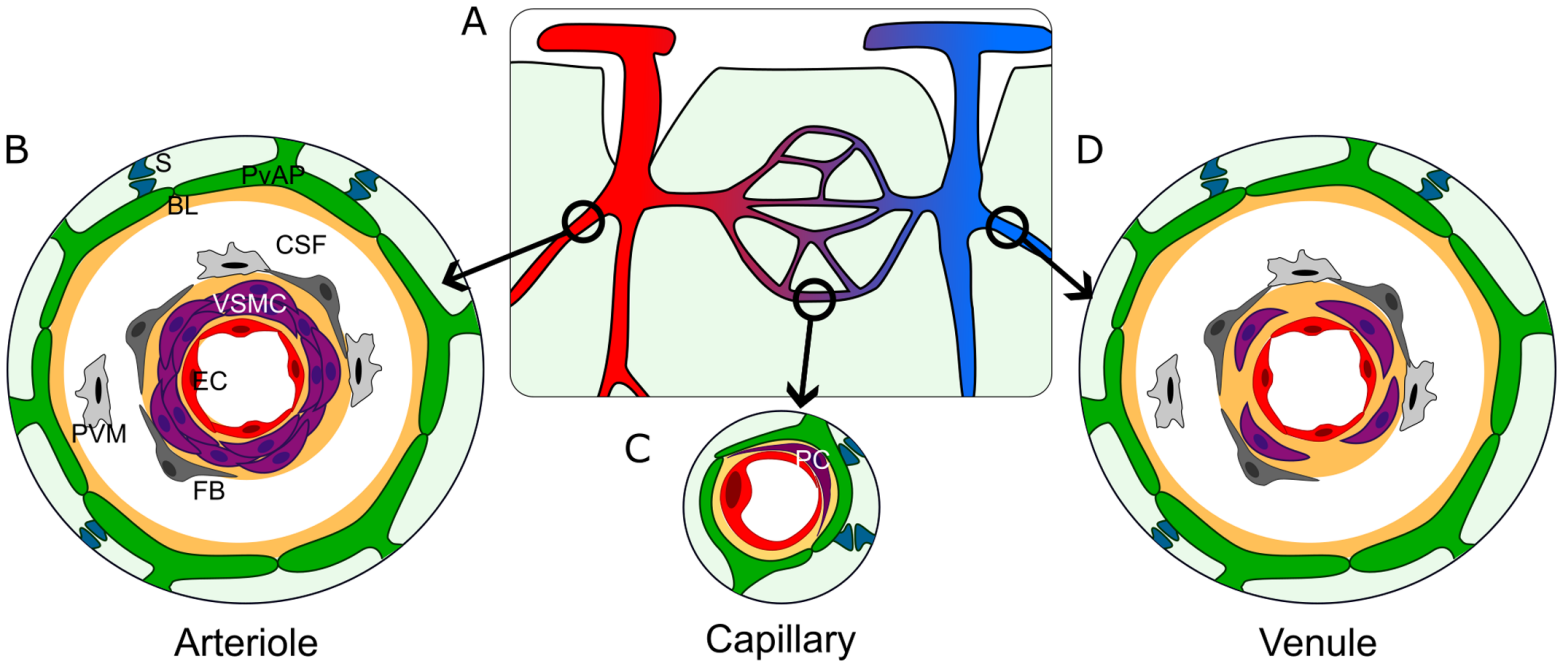
Architecture of the gliovascular interface in the central nervous system. **(A)** Schematic representation of the brain vasculature. Arterioles (red); capillaries (purple); venules (blue). **(B–D)** Organization of the gliovascular interface around arterioles **(B)**, capillaries **(C)** and venules **(D)**. Perivascular astrocyte processes (green, PvAP); perivascular synapses (dark blue, S); BL (yellow, BL); Cerebrospinal fluid (white, CSF); perivascular macrophages (light gray, PVM); fibroblasts (dark gray, FB); vascular smooth muscle cells (purple, VSMC; **B–D**) or pericytes (purple, PC; **C**); endothelial cells (red, EC).

Gliovascular organization varies along the vascular tree (Lendahl et al., 2019; Figure 1). At the brain surface, PvAPs form the *glia limitans* (GL), which is separated from the pial vessels, arteries, and veins by a space filled with CSF. The CSF-filled space surrounding penetrating arteries and arterioles and exiting veins and venules is called the Virchow-Robin space. In deeper cortical layers, this space gradually narrows until disappearing at the capillary level where PvAPs contact the BVs (Figure 1). This structural diversity also corresponds to a variety of cellular interactions. The CSF contains various type of immune cells (Rebejac et al., 2024). The endothelial walls of arteries, veins, large arterioles and venules are surrounded by VSMCs, fibroblasts, and perivascular macrophages (PVMs; Karam et al., 2022), while capillary endothelial walls are only enwrapped by pericytes. Notably, the endothelial and mural cells (VSMCs and pericytes) are surrounded by an extracellular matrix layer, known as the BL. Astrocytes also contribute to the BL, making it doubly layered around large vessels that are bathed in CSF, while at the capillary level, endothelial cells, pericytes and astrocytes participate to the formation of a unique BL (Figure 1). Interestingly, 3D EM reconstruction of brain vessels showed that the structure of PvAPs in the cortex vary in thickness and organization, being more cord-like principal processes around vessels close to the pial suface (Watanabe et al., 2010). PvAPs surrounding large vessels have bigger individual surface areas than on capillaries (Wang et al., 2021). As mentioned earlier, while astrocytes contact relatively few vessels, they encompass thousands of synapses. Interestingly, unlike PAPs which are mostly purely neuroglial contacts, PvAPs also interact with synapses on the parenchymal side (Cohen et al., 1995; Boulay et al., 2017; Mazaré et al., 2020; Figure 1). Thus, the simplified representation of astrocytes as being “quartered” between neurons and BV is untrue. The subcellular organization of PvAPs is complex. PvAPs contain mitochondria (Mathiisen et al., 2010; Gӧbel et al., 2020) and rough and smooth endoplasmic reticulum (ER; Boulay et al., 2017). Seven percent of them contain Golgi outposts, suggesting a role in local protein maturation and secretion (Boulay et al., 2017). Additionally, polysomes (multiple ribosomes simultaneously translating a single mRNA) are also present, indicating that local translation is active in this compartment (Boulay et al., 2017; Avila-Gutierrez et al., 2024). At the molecular level, PvAPs are highly specialized domains, enriched with several key proteins (see the next chapter; Cohen-Salmon et al., 2020; Díaz-Castro et al., 2023).

### 2.1 Summary

Astrocytes are highly branched glial cells that form complex interactions with neurons and blood vessels. Their characteristic star-like shape primarily reflects the organization of their intermediate filament cytoskeleton. Astrocytes form intricate bushy processes that define specific territories containing numerous synapses. Astrocytes also contact BVs through PvAPs, a term we prefer over “endfeet” to better reflect the diversity and complexity of these contacts. Astrocytes provide nearly complete vascular coverage, interrupted only by rare discontinuities where neurons, oligodendrocytes, or microglia directly contact BVs. Astrocyte–BV interactions vary across brain regions, involving diverse structural and cellular components, including immune cells in the CSF, PVMs, fibroblasts, VSMCs around arteries, arterioles, venules, and veins, as well as pericytes around capillaries. Notably, these contacts are always indirect, with cells separated by the BL—a structure that coordinates intercellular communication—and/or the CSF-filled compartment. PvAPs exhibit a complex subcellular architecture, containing mitochondria, ER and Golgi apparatus, along with a distinct molecular repertoire, including proteins that are locally translated.

### 2.2 Some open questions

- The term ‘endfeet’ is insufficient to fully defines astrocyte-vascular contact diversity, as the literature suggests—without specifically addressing this point— that they do not always form a unique and large “foot” but may rather exhibit various morphologies in terms of size and shape. In fact, the organization of PvAPs remains poorly understood. A more comprehensive approach is needed, one that includes the observation of contiguous astrocytes at different vascular levels.
- The organization and role of synapses abutting PvAPs have yet to be explored (Cohen et al., 1995; Boulay et al., 2017; Mazaré et al., 2020). This synaptic arrangement may exhibit diversity—for example, in the balance between inhibitory and excitatory inputs—introducing an additional layer of heterogeneity among PvAPs. What types of synapses are involved? Do these synaptic features differ from those found on non-PvAPs? These compelling questions open new avenues for investigation in the field.
- The heterogeneity of astrocytes should be considered in future studies, as it likely contributes to PvAP diversity (Batiuk et al., 2020; Endo et al., 2022; Bocchi et al., 2025).
- The subcellular organization of PvAPs is highly heterogeneous, indicating a diverse range of properties and functions that remain largely unexplored. For instance, do the 7% of PvAPs containing a Golgi apparatus play a specialized role in the maturation and secretion of specific proteins? Furthermore, are all proteins secreted by PvAPs subjected to identical post-translational modifications?

## 3 Development of PvAPs and their molecular identity

In this section, we summarize current knowledge on the timing of PvAP formation and the acquisition of their specific molecular repertoire with a focus on key proteins.

**Figure 2:**
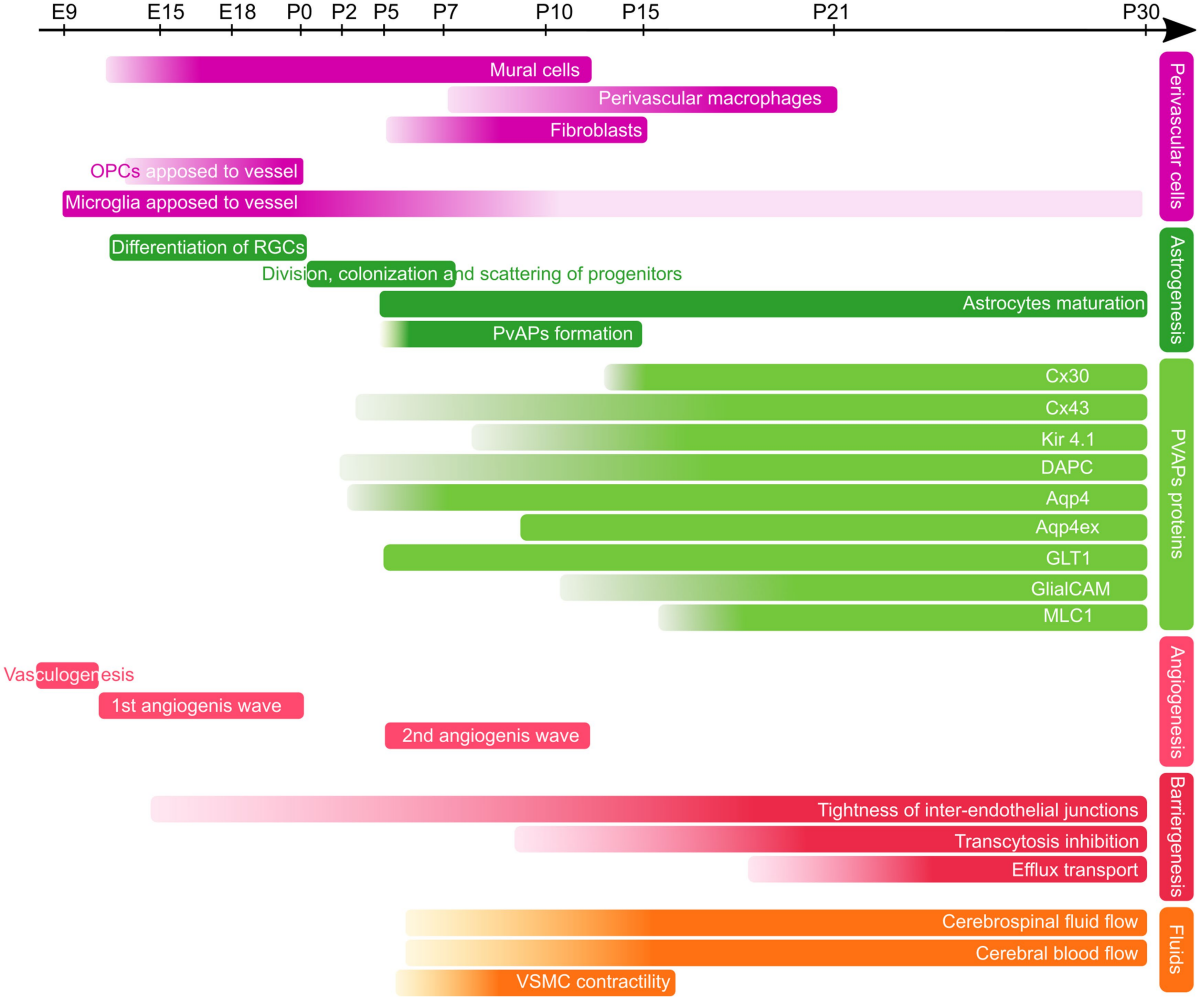
Developmental timeline of PvAPs and associated key proteins in the mouse brain. Perivascular cells (purple), Astrogenesis and PvAP-associated proteins (green), Angiogenesis and Barriergenesis (red), Fluids (orange); OPCs, Oligodendrocyte Progenitor Cells, RGCs, Radial Glial Cells; PvAps, Perivascular Astrocyte Processes; Cx, Connexin; DAPC, dystrophin-associated protein complex; Aqp4, Aquaporin 4; GlialCAM, Glial Gell Adhesion Molecule; MLC1, Megalencephalic leukoencephalopathy with subcortical cysts-1; VSMC, Vascular smooth Muscle Cell. E, Embryonic day; P, Postnatal day.

In the mouse cortex, astrocytes progenitors, together with postnatal OPCs, are generated from late radial glial cells after completion of neurogenesis. They first follow a proliferation phase during the week before and after birth (Figure 2). This proliferation phase is followed by differentiation and maturation of astrocytes until around postnatal day 21 (P21; Clavreul et al., 2019). In humans, astrogenesis begins around gestational week (GW) 16–18 and continues after birth until the brain is fully tiled and the vasculature completely covered (de Majo et al., 2020; Degl’Innocenti and Dell’Anno, 2023).

A key question is how astrocyte progenitors develop processes around BVs. Recent findings suggest that multiple mechanisms are involved. Freitas-Andrade et al. observed that at birth, many astrocyte progenitors are primarily located closed to BVs, with their processes aligned along BVs (Freitas-Andrade et al., 2023). By P5, however, their cell bodies appear to migrate away from the vasculature (Freitas-Andrade et al., 2023). Cell soma initially positioned on BVs could thus separate from the vascular compartment while maintaining contacts, rather than extending distal processes from a parenchymal position. At P5, TEM observations also revealed that PvAPs already contained Golgi outposts, polysomes and ER cisternae, indicating early proteostasis within PvAPs (Avila-Gutierrez et al., 2024). Between P5 and P10, astrocyte vascular coverage increases rapidly and is almost complete by P15 (Mondo et al., 2020; Gilbert et al., 2021; Freitas-Andrade et al., 2023; Avila-Gutierrez et al., 2024; Figure 2). However, this timeline may vary across brain regions, as dorso-ventral differences in astrocyte maturation have been observed (Freeman, 2010). Interestingly, microglia have been shown to migrate along BVs during cortical development with their number progressively decreasing from P1 to P21. This reduction coincides with the gradual extension of astrocyte vascular coverage (Mondo et al., 2020; Figure 2). Other cell types, such as oligodendrocyte precursors (Tsai et al., 2016) or neuroblasts (Bovetti et al., 2007; Whitman et al., 2009), migrate along BVs during brain development. Whether these cells interact with astrocytes to regulate the formation of PvAPs remains unknown.

Aquaporin 4 (Aqp4) is a bidirectional water channel enriched in PvAPs and implicated in water perivascular homeostasis and drainage (see chapter 4). It is expressed throughout the CNS and around all vessel types (Nielsen et al., 1997). Aqp4 in astrocyte perivascular membranes form functional tetramers arranged into orthogonal arrays of particles (OAPs; Dermietzel, 1973; Wolburg, 1995; Furman et al., 2003). Most OAPs are composed of two Aqp4 isoforms, AQP4a (M1: 32 kDa) and AQP4c (M23: 30 kDa), transcribed from different initiating methionine (Furman et al., 2003). *In vitro*, M23 isoform is essential to OAP formation (Crane and Verkman, 2009). Other cytoplasmic isoforms have been described *in vitro* (Lisjak et al., 2017, 2020). Aqp4 expression begins around birth in mice, before birth in pigs, and between gestational weeks 19 and 22 in humans (El-Khoury et al., 2006; Fallier-Becker et al., 2014; Sobierajski et al., 2024). The maturation of Aqp4 and its anchorage in perivascular astrocytic membranes are heterogeneous. We have shown that Aqp4 expression in PvAPs develops progressively until P5, at which point it is detected in all PvAPs (Ezan et al., 2012; Gilbert et al., 2019; Figure 2). Using GVU isolation in which PvAPs stay attached to BVs, we found that Aqp4 level increased from P5 to P15 (Avila-Gutierrez et al., 2024). Another study reported that Aqp4 was undetectable at birth and appeared at P4 with stronger signals in the pial *glia limitans* and continued to accumulate until P21 in PvAPs and until P13 in the *glia limitans* (Lunde et al., 2015). In adults, Aqp4 expression is higher in areas with reduced extracellular space (Badaut et al., 2002). Recent studies revealed the existence of Aqp4ex isoforms (M1ex, 38 kDa; and M23ex, 35 kDa) in which a 29-amino-acid C-terminal extension is generated by translational readthrough (Bellis et al., 2017; Palazzo et al., 2019; Sapkota et al., 2019). They represent about 10% of all Aqp4. Aqp4ex regulates *α*-syntrophin localization in PvAPs (Mueller et al., 2023). Inactivation of Aqp4ex leads to mislocalizaton of Aqp4 which redistributes away from the perivascular membrane (Bellis et al., 2017; Palazzo et al., 2019). The phosphorylation of Aqp4ex C-terminal tail regulates the water channel gating as well as the vesicular trafficking of Aqp4 to the plasma membrane (Bellis et al., 2017). Aqp4ex is predominantly localized to PvAPs, with a weaker expression observed in the *glia limitans* (Palazzo et al., 2019). Interestingly, Aqp4ex expression begins several days after Aqp4 as it is not detected in P9 brain extracts. This suggests that Aqp4 water channel activity is not fully mature at this stage (Sapkota et al., 2019; Figure 2). Mechanisms regulating Aqp4ex expression are yet unknown.

Mlc1 and GlialCAM account for megalencephalic leukoencephalopathy with subcortical cysts-1 and Glial Cell Adhesion Molecule, respectively. Their absence leads to Megalencephalic leukoencephalopathy with subcortical cysts (MLC), a rare type of leukodystrophy (Passchier et al., 2024; see chapter 5). Together, they form a membrane junctional complex between PvAPs (Teijido et al., 2007; López-Hernández et al., 2011; Hoegg-Beiler et al., 2014). The presence of Mlc1 in PvAPs membranes is necessary for the membrane anchorage of GlialCAM, and vice versa (Hoegg-Beiler et al., 2014). Mlc1 is expressed only by astrocytes in the CNS, while GlialCAM is also expressed by oligodendrocytes. Both are strongly enriched in PvAPs and are more present at the level of large BV than capillaries (Wang et al., 2021). GlialCAM expression precedes Mlc1 in the PvAPs at P5, with both expressions increasing between P10 and P15 (Gilbert et al., 2019; Figure 2). Absence of Mlc1 in the mouse alters the development of the astrocyte vascular coverage and several vascular functions, as discussed in the chapter 4. Interestingly, the absence of the Mlc1/GlialCAM complex results in vascular areas being abnormally devoid of PvAPs and contacted by axons. This suggests that axons and PvAPs may compete for access to the surface of blood vessels.

The BL is an extracellular matrix layer surrounding all brain BVs. It is formed by vascular cells and astrocytes and is crucial to the BBB integrity. BL contributed by PvAPs is in contact either with the CSF in the Virchow-Robin space or fused with BL surrounding capillaries (Figure 1). Laminins are major components of the BL and are ligands for integrins (Sixt et al., 2001; del Zoppo and Milner, 2006; Milner et al., 2008; Venkatesan et al., 2015) and the dystroglycan complex (Moore et al., 2002). Laminins form hetero-tetramers of alpha, beta and gamma chains. Astrocytes secrete laminin 211. Both laminins and integrins establish intricated combinations as endothelial cells, astrocytes and mural cells express different isoforms (reviewed in Halder et al., 2023; Nasrollahi and Yao, 2025). Expression of laminins and their receptors is upregulated during development but their specific developmental profile in astrocytes is unclear. In addition to laminins, astrocytes express several other BL components: collagen IV which plays an essential role in maintaining the laminin structure (Gould et al., 2005), perlecan, agrin and fibronectin (del Zoppo and Milner, 2006; Farach-Carson et al., 2014).

The dystrophin-associated protein complex (DAPC) is composed of the dystrophin isoform Dp71, *α*-dystrobrevin-1, α1-syntrophin, and *β*-and α-dystroglycan (Culligan and Ohlendieck, 2002; Heuser et al., 2012; Belmaati Cherkaoui et al., 2021). It links the cytoskeleton to laminin and agrin in the BL (Gee et al., 1994; Noell et al., 2011; Wolburg et al., 2011). α-syntrophin is an adaptor protein of the DAPC. It is present at higher densities in astrocyte membrane domains facing pericytes (Gundersen et al., 2014). Its depletion causes the almost complete loss of Aqp4 in PvAPs (Neely et al., 2001; Hoddevik et al., 2017, 2020). α-syntrophin appears only around P13 in mouse cortical PvAPs (Ezan et al., 2012; Lunde et al., 2015; Figure 2). Similarly, β-dystroglycan is only detectable in PvAPs from P7 (Lunde et al., 2015). Dp71 expression profile was analyzed in the rat hypothalamic supraoptic nucleus, where it first appears at birth and progressively increases until P60 (Souttou et al., 2019).

Kir4.1 is an inward rectifying K^+^ channel expressed by astrocytes and oligodendrocytes (Kalsi et al., 2004). In astrocytes, its expression is highly enriched in PvAPs (Higashi et al., 2001) but it is also present in perisynaptic processes where it allows uptaking the excess of extracellular K^+^ released by synapses (Sibille et al., 2014; Sibille et al., 2015). Whether perivascular Kir4.1 has the same role is unclear. It might buffer K^+^ released by perivascular synapses. Depending on K^+^ gradient, it could also release K^+^ at the perivascular level away from active synapses. Indeed, Kir4.1 can contribute to an outward K^+^ flux as shown in the inner ear *stria vascularis* (Marcus et al., 2002). Kir4.1 is weakly detected at P7 in PvAPs and its level progressively increases until P60 (Avila-Gutierrez et al., 2024; Figure 2). A gradual shift in Kir4.1 localization, from the soma to the distal processes, occurs during postnatal development (Moroni et al., 2015).

GLT1 is a sodium-dependent glutamate transporter specifically expressed in astrocytes in the brain (Kim et al., 2011). Like Kir4.1, it is highly present in PvAPs where it recaptures excess of glutamate released by neurons at the synapse upon neurotransmission. It is expressed in PvAPs only from P10 and highly enriched from P15 (Avila-Gutierrez et al., 2024) but its specific role in PvAPs is not known (Figure 2).

Astrocytes are highly interconnected cells which form extensive networks through gap junction channels composed of connexin 30 (Cx30) and connexin 43 (Cx43; Giaume et al., 2010). Both proteins are highly enriched in PvAPs and likely contribute to the strong coupling observed between PvAPs (Rouach et al., 2008; Ezan et al., 2012). In addition to their gap junction functions, both connexins form hemichannels allowing a direct communication between the cytoplasm and the extracellular space, and exert non-channel functions related to their interactions with intracellular proteins (Giaume et al., 2013; Pannasch and Rouach, 2013; Van Campenhout et al., 2020). At the vascular interface, the absence of Cx30 leads to the upregulation of *γ*-sarcoglycan, which is part of the smooth muscle sarcoglycan complex (Boulay et al., 2015b). In mice, the expression of Cx30 begins in astrocytes at P10 and the protein concentrates at the perivascular level after P12 (Ezan et al., 2012; Figure 2). Cx43 is expressed from the onset of astrogenesis but accumulates in PvAPs around large BV from P2, and around capillaries only from P10 (Ezan et al., 2012; Figure 2). Cx43 regulates immune quiescence; its absence results in endothelial reactivity, BBB weakness (opening upon shear stress), recruitment of peripheral immune cells and the development of an autoimmunity against the von Willebrand a5a (Vwa5a), an extracellular matrix protein present around astrocyte somata (Boulay et al., 2015a). Between P10 and P15, an increase in Cx43 expression is observed in PvAPs and its level remains stable thereafter (Ezan et al., 2012; Gilbert et al., 2019). The loss of Cx43 leads to a breakdown in immune quiescence beginning in late postnatal development (around P25), marked by increased astrocytic cytokine expression and peaking immune cell recruitment at approximately 6 weeks (Boulay et al., 2015a).

### 3.1 Summary

In mice, PvAPs begin to develop around birth. By postnatal day 15 (P15), they achieve near-complete coverage of the vasculature, though this timeline may vary across brain regions. During this developmental period, the number of perivascular migrating microglia and neural precursor cells declines. They are replaced by PvAPs. Concurrently, PvAPs acquire a specific molecular repertoire associated with key gliovascular functions, including BBB properties, fluid drainage, immune regulation, and perivascular homeostasis.

### 3.2 Some open questions

- Aqp4 labeling is used in many studies as a marker of PvAPs formation, while PvAPs form in the absence of Aqp4 (Haj-Yasein et al., 2011). It is therefore necessary to reconsider the use of Aqp4 as an exclusive marker of PvAP maturation. In this context, greater emphasis should be placed on employing astrocyte-specific cytoplasmic or membrane-targeted fluorescent reporters, as well as quantitative ultrastructural approaches, to more accurately study the development of PvAPs.
- The organization of the dialog between migrating cells along blood vessels and astrocytes during postnatal development remains largely unexplored. Investigating the localization of microglia and oligodendrocytes in conditions where astrocytic coverage is abnormal or incomplete—such as in Mlc1 or Hmgb1 knockout (KO) mice—could provide insight into whether these cell types compete for access to the vascular surface during development.
- Our understanding of the PvAP molecular repertoire remains limited. To advance this knowledge, innovative approaches—such as characterizing local translation profiles or local proteomes—are essential (Boulay et al., 2017; Avila-Gutierrez et al., 2024; Soto et al., 2024).
- The delayed expression of Aqp4ex and *α*-syntrophin—both essential for the assembly of Aqp4 channels in PvAPs membranes—suggests that water exchange and drainage mechanisms do not reach maturity before at least P12 in mice.
- The mechanisms enabling ribosome readthrough and the synthesis of Aqp4ex remain largely unexplored. Such mechanisms could also facilitate the generation of alternative isoforms of PvAP proteins, as suggested for Mlc1 by ribosome profiling RNA-seq data in astrocytes (Sapkota et al., 2019).
- The developmental profile of extracellular matrix proteins secreted by astrocytes is unclear.

## 4 Molecular mechanisms for PvAP development

While the literature on astrocyte molecular and morphological development is growing, the formation of the astrocyte-vascular interface is rarely addressed. In this chapter, we will list the few known mechanisms and suggest some interesting candidates.

In the mouse, the development of PvAPs is postnatal and coincides with many important developmental processes: a postnatal angiogenic wave during which capillary volume doubles (Coelho-Santos and Shih, 2020; Coelho-Santos et al., 2021); synaptogenesis and synaptic refinement (Stogsdill et al., 2017); arterial VSMC maturation (Slaoui et al., 2023); recruitment of PVMs and fibroblasts (Dorrier et al., 2022; Karam et al., 2022; Jones et al., 2023); postnatal maturation of the BBB; detachment of perivascular microglia (Mondo et al., 2020). Therefore, it is likely that, in addition to intrinsic mechanisms, proliferating and migrating astrocytes sense and respond to extrinsic signaling cues from various cellular components of the brain, to develop vascular contacts and ensure that PvAPs are correctly positioned and mature into functional compartments.

Postnatal angiogenesis could influence astrocyte differentiation via a modulation of the oxygen level, which increases locally with the formation of new vessels and the expansion of perfusion. In the retina, enhanced oxygen blocks astrocyte proliferation and promotes their differentiation, via the upregulation of GFAP (West et al., 2005). Oxygen also suppresses the expression of the transcription factor *tailless* Tlx, enabling a phenotypic switch of retinal astrocytes from proangiogenic immature to mature astrocytes (Uemura et al., 2006). In contrast, leukemia inhibitory factor (LIF), which is predominantly expressed in the developing endothelium and whose receptor is expressed in retinal astrocytes maintains the proangiogenic immature astrocyte phenotype (Kubota et al., 2008). These examples of gliovascular developmental crosstalk are based on the retina but similar mechanisms may occur in the brain.

Fibroblast growth factors (FGFs) represent another family of growth factors with potential autocrine and paracrine effects on astrocyte differentiation. Complete inactivation of FGF2 or FGF5 affects GFAP expression in PvAPs (Reuss et al., 2003). In the single FGF2 knockout mouse model, PvAP development is delayed (Saunders et al., 2016).

Mlc1 and GlialCAM form an astrocyte-specific adhesion complex between PvAPs. Their expression in PvAPs starts around P5 and they gradually form a network between PvAPs which becomes mature by P15 (Gilbert et al., 2019). This developmental process correlates with the progressive formation of astrocyte vascular coverage between P5 and P15 (Gilbert et al., 2021; see previous chapter). In *Mlc1* KO mice, GlialCAM is no longer stabilized in astrocyte membranes, resulting in the complete absence of the complex (Hoegg-Beiler et al., 2014). In this condition, astrocytes fail to polarize properly, and PvAPs development is altered (Gilbert et al., 2021). PvAPs loose also they adhesiveness to the vascular surface (Gilbert et al., 2021). The main known consequence of this rearrangement is not BBB leakage, but rather incomplete VSMC postnatal differentiation toward contractile phenotype, indicating an as-yet-unknown signaling pathway between astrocytes and VSMCs (Gilbert et al., 2021; Slaoui et al., 2023). Of note, the potential relationship between PvAPs and VSMCs was already highlighted in a mouse model in which laminin γ1 was inactivated in astrocytes and resulted in a reduced level of contractile VSMC proteins like smoothelin, smooth muscle actin and SM22 (Chen et al., 2013).

The high mobility group box 1 protein (HMGB1) is a chromatin-associated protein. Inactivation of HMGB1 in astrocytes impairs astrocyte morphology, including PvAP formation. A partial loss of vascular coverage together with altered polarized expression of PvAP proteins such as Aqp4 and Cx43 were reported (Freitas-Andrade et al., 2023). Astrocyte-specific deletion of HMGB1 also has a strong effect on endothelial cell morphology with development of intraluminar membrane extensions and vacuoles with however no effect on the BBB integrity (Freitas-Andrade et al., 2023).

PDGF-*β* is expressed by endothelial cells and is a crucial growth factor for the recruitment of pericytes and VSMCs, as well as the development of the BBB (Hellstrom et al., 1999; Daneman et al., 2010). Recent studies have shown that PDGF-β is essential for the perivascular polarity of Aqp4 (Daneman et al., 2010; Munk et al., 2019). However, the loss of Aqp4 polarity observed in this pericyte-deficient mouse model might also result from early vascular malformations and BBB disruption.

BL formation is probably a key element in the development of PvAPs. Indeed, deletion of laminin α2 (expressed by both pericytes and astrocytes) or astrocytic laminin γ1 alters Aqp4 localization in PvAPs and pericyte differentiation (Menezes et al., 2014; Yao et al., 2014). Lack or astrocytic laminin γ1 alters BBB integrity by 6 months of age at the level of small arterioles located in deep regions of the brain (Chen et al., 2013). As discussed above, it also affects the level of contractile VSMC proteins which could deregulate the cerebral blood flow and neurovascular coupling (Chen et al., 2013). Astrocyte integrin β1, via its binding to laminin α4, regulates the formation of PvAP (Segarra et al., 2018). Astrocyte dystroglycan is crucial to Aqp4 localization in PvAPs (Moore et al., 2002; Noell et al., 2011). The DAPC is also essential for the membrane anchorage of Aqp4 and the potassium channel Kir4.1 (Neely et al., 2001; Connors et al., 2004; Hoddevik et al., 2017). Absence of fibronectin alters PvAPs formation (Liu et al., 2023).

Local translation is a common mechanism in morphologically complex cells and organizes cell compartmentation. It allows a rapid response to local needs of specific proteins without requiring protein trafficking. In neurons, it is a key mechanism for axon development and guidance (Jung and Holt, 2011). Astrocytes display local translation in PvAPs (Boulay et al., 2017). In a recent study, local translation was detected in newly formed PvAPs at P5 (Avila-Gutierrez et al., 2024). This study revealed that mRNA and polysomal mRNA levels in PvAPs can exhibit distinct developmental profiles. For genes such as Glt-1, Mlc1, and Kir4.1, protein expression levels in PvAPs mirrored the developmental patterns observed for both total and polysomal mRNAs. In contrast, no such correlation was observed for Aqp4. These observations suggest that mixed regulatory mechanisms likely govern the developmental expression of proteins in PvAPs and that local translation may be key for newly generated astrocytes to support the formation and differentiation of PvAPs (Avila-Gutierrez et al., 2024).

Finally, microglia have been shown to migrate along BVs during cortical postnatal development with their numbers progressively decreasing from postnatal day 1 (P1) to P21—coinciding with the gradual formation of astrocytic vascular coverage (Mondo et al., 2020). Similarly, BVs serve as a migration route for oligodendrocyte precursors and neural stem cells (Bovetti et al., 2007; Whitman et al., 2009; Tsai et al., 2016; Fujioka et al., 2019). Whether an interplay between these cells and astrocytes exists to regulate the formation of PvAPs remains unknown.

### 4.1 Summary

The development of PvAPs is a postnatal event shaped by multiple factors, yet the underlying mechanisms remain unclear. PvAPs emerge in parallel with key developmental processes such as angiogenesis, synaptogenesis, and blood–brain barrier (BBB) maturation. Extrinsic cues from the vascular environment—including oxygen availability and growth factors—play a critical role in astrocyte differentiation and may regulate the formation of PvAPs. Proteins such as MLC1, GlialCAM, and HMGB1 are implicated in establishing astrocyte polarity and promoting PvAP adhesion to the vascular surface. In turn, the maturation of PvAPs can influence vascular development, including the differentiation of endothelial cells and VSMCs. Additionally, local translation within astrocytes may support the structural and functional differentiation of PvAPs.

### 4.2 Some open questions

- PvAPs formation might be orchestrated by guidance and/or repulsive cues. Combined omics characterization of vascular cells and astrocytes at crucial steps of PvAP development could help to identify them. For example, it could be valuable to investigate the compatibility of neuro-glio-vascular receptor-ligand interactions during development.
- The exact mechanisms underlying the initiation of PvAP formation remain unclear. Some astrocytes may extend their processes toward blood vessels (BVs), while others might form PvAPs by retracting from the BV, yet maintaining a connection with the vascular surface.
- PvAPs could be motile elements as suggested by recent studies showing that PvAPs can grow back after laser ablation (Kubotera et al., 2019; Mills et al., 2022).
- Mechanisms regulating the developmental switch between astrocytes and microglia at the capillary surface are yet unknown.
- Does local translation in PvAPs participate to their formation, and if so, how?
- Given that the absence of the MLC1/GlialCAM adhesion complex between PvAPs results in axon insertion at the endothelial surface, do astrocytes and neurons compete for perivascular space, or do they coordinate their activities during postnatal development to establish a well-organized and functional neuro-glio-vascular interface?

## 5 Developmental roles of PvAPs

In this chapter, we will concentrate on how astrocytes and PvAPs influence the development of some crucial vascular functions.

### 5.1 Angiogenesis and barrier-genesis

Integrity of the BBB relies primarily on EC properties: (i) Tight junctions and *adherens* junctions restricting the paracellular passage and the low rate of transcytosis, thereby limiting the paracellular and transcellular passage respectively; (ii) Transporters of the SLC family and ATP-binding cassette pumps for selective influx/efflux of molecules; and (iii) The BL, which blocks immune infiltration, anchors cells of the GVU and binds signaling molecules. BBB integrity is induced embryonically in the mouse brain by pericytes, which are recruited on newly formed BVs from E10, induce tight junction formation and restrict transcytosis (Daneman et al., 2010; Biswas et al., 2020). Early *in vitro* observations suggested a role for astrocytes in BBB integrity (Igarashi et al., 1999). However, it pertains primarily to postnatal maturation or maintenance mechanisms and not to BBB development *per se* since astrocytes are generated perinatally.

A major regulator of BBB induction and maintenance is the Wnt/*β*-catenin pathway and in particular the ligands Wnt7a/7b (Liebner et al., 2008; Stenman et al., 2008; Daneman et al., 2009; Zhou et al., 2014; Blanchette and Daneman, 2015; Vanhollebeke et al., 2015). Astrocytes have been recently shown to express the non-canonical Wnt ligands Wnt4, Wnt5a and Wnt11 (Guérit et al., 2021). The early deletion in astrocytes of *evenness interrupted* (*Evi*) encoding the Wnt secretion mediator was recently shown to have no effect on brain vascular development but on the maintenance of the BBB from 10 weeks of age (Guérit et al., 2021). Interestingly, endothelial cells did not exhibit any defects in the expression of genes classically associated with BBB integrity, such as claudin-5. Instead, they showed elevated levels of Cav1, suggesting an increased rate of transcytosis. Additionally, swelling of PvAPs was observed, along with impaired localization of Aqp4 in astrocytic perivascular membranes. These findings indicate that astrocyte-derived Wnt signaling does not influence angiogenesis or BBB formation, but is essential for the maintenance of BBB integrity.

Norrin is a growth factor of the TGFβ superfamily. Upon activation, this receptor signals via β-catenin to activate LEF/TCF-mediated transcription in an LRP5/6-dependent manner (Ye et al., 2009; Wang et al., 2012). Norrin is expressed by a subset of astrocytes as early as P1 and could participate to this signalization (Miller et al., 2019; Rurak et al., 2022).

As described previously, the BL forms progressively during embryonic and postnatal development. BL serves both as a physical barrier and as a signalization platform for molecules such as Pdgfβ, VEGF or TGF-β, that bind BL components and eventually form gradients. Astrocytes contribution to the BL during development participates to the regulation of BBB maturation and maintenance. Collagen IV is produced by all cells of the GVU at a constant level during postnatal development (Baeten and Akassoglou, 2011; Slaoui et al., 2023). It is crucial for the assembly of the BL. Loss of the pro-collagen type IV alpha 1 due to mutations in *Col4a1* causes perinatal cerebral hemorrhage and porencephaly (Gould et al., 2005). Deletion of the laminin α2 chain gene (*Lama2*; Menezes et al., 2014) or astrocyte-specific deletion of the laminin γ1 chain (*Lamc1*; Chen et al., 2013; Yao et al., 2014) leads to the absence of laminin 211 formed by astrocytes. It induces BBB defects due to the loss of pericytes and the disorganization of tight junctions. Deletion of either dystroglycan (DAG1KO) or integrin β1 leads to similar phenotypes (Menezes et al., 2014). Integrin αv deletion in glial cells (but not in endothelial cells or in pericytes) leads to transitory hemorrhages in late embryonic and early postnatal stages (McCarty et al., 2005). Astrocyte integrin αvβ8 activates TGF-β regulating BV development (Cambier et al., 2005).

As previously discussed, astrocytes PvAPs are coupled via gap junctions composed of Cx30 and Cx43. Combining the full KO of Cx30 and the early deletion of Cx43 specifically in astrocytes, results in a loosening of the BBB which is leaky only when a shear stress is applied at the luminal surface. Thus, as for Wnt, astrocytic connexins are important to maintain BBB. In the single *Cx43* KO, astrocytes and endothelial cells become reactive leading to the abnormal recruitment of peripheral immune cells and the elaboration of a humoral response against astrocytes (Boulay et al., 2015a; Boulay et al., 2016; Boulay et al., 2018). No BBB phenotype is detected in mice deleted for Cx30 (Boulay et al., 2015b). Thus, only Cx43 regulates BBB maintenance and immune quiescence.

### 5.2 CSF circulation development

More than a decade ago, the glymphatic system—a global circuit responsible for CSF and ISF drainage—was described. This system facilitates the clearance of metabolic waste products, including proteins such as amyloid β (Iliff et al., 2012). Together with the identification of the meningeal lymphatic network (Aspelund et al., 2015; Louveau et al., 2015) and the proposal of alternative models for intra-parenchymal CSF circulation (Lendahl et al., 2019), this discovery helped establish a new paradigm for brain–immune system interactions. The proper function of the glymphatic system appears to rely on the presence of Aqp4 in PvAPs. Notably, the maturation of the glymphatic system follows the perivascular polarization of Aqp4 (Munk et al., 2019). It is impaired in *Aqp4* KO models (Mestre et al., 2018a; Abbrescia et al., 2024), and in the pericyte-deficient model, where Aqp4 polarization in PvAPs is disrupted (Munk et al., 2019). The postnatal formation of complete vascular coverage by PvAPs may itself promote glymphatic flow by establishing a conduit around large blood vessels to guide fluid movement (Plog et al., 2018). Interestingly, in *Mlc1* KO mice, where vascular coverage is incomplete, CSF circulation is impaired despite preserved Aqp4 polarity (Gilbert et al., 2021). In this case, the reduced glymphatic activity may instead result from altered arterial contractility. Indeed, CSF flow within perivascular spaces is directly influenced by fluctuations in blood pressure along vessel walls (Mestre et al., 2018b). The shear stress generated by CSF flow could be sensed by astrocytic mechanoreceptors, potentially enabling astrocytes to regulate CSF dynamics in response. This concept is supported by a recent *in vitro* study showing that sphingosine-1-phosphate (S1P) can activate the mechanosensitive channel Piezo1 in primary astrocytes, leading to calcium influx (Cibelli et al., 2024).

### 5.3 Cerebral blood flow and neurovascular coupling development

The brain accounts for 20% of total body energy consumption, and tight coordination between neuronal activity and cerebral blood flow is essential to meet the high metabolic demands of neurons (Attwell et al., 2010). In the mouse, the basal CBF is very low at P5 and increases during postnatal development. This phenomenon spatially correlates with increasing VSMC contractility (Slaoui et al., 2023) and the postnatal angiogenic phase (Coelho-Santos and Shih, 2020; Coelho-Santos et al., 2021). This process is impaired in the absence of Mlc1 (Gilbert et al., 2021). Absence of astrocytic laminin γ1 also affects the expression of VSMC contractile proteins such as SMA, smoothelin or SM22 (Chen et al., 2013). Thus PvAPs development is crucial to the maturation of VSMCs and therefore to the development of the CBF. In the mature brain, astrocytes play a crucial role in regulating the neurovascular coupling (Mishra et al., 2016). Synaptic neurotransmitter release triggers an increase in intracellular Ca^2+^ levels in astrocytic processes and the soma. In PvAPs, these Ca^2+^ transients are followed by the release of vasoactive molecules at the vascular interface, leading to either dilation or constriction of the BVs (Zonta et al., 2003; Mulligan and MacVicar, 2004; Otsu et al., 2015; Lind et al., 2018). A major mechanism is the role of arachidonic acid (AA) metabolites, which are generated by sequential action of phospholipases A2 and D2 and COX1, both enzymes being induced by Ca^2+^ increase (Mishra et al., 2016). This results in the production of prostaglandin E2 (PGE2), epoxyeicosatrienoic acids (EETs) or 20-hydroxyeicosatetraenoic acid (20-HETE). PGE2 liberation at the vascular interface induces the dilation of cortical arterioles (Takano et al., 2006), as EETs (Peng et al., 2002; Metea and Newman, 2006). In contrast, 20-HETE is a potent vasoconstrictor that might serve as a negative feedback mechanism to prevent overperfusion (Mulligan and MacVicar, 2004). Ca^2+^ elevation in astrocytes can also result in the activation of large-conductance K^+^ (BK) channels expressed in PvAPs which results in K^+^ efflux onto BVs leading to their dilation or constriction (Price et al., 2002; Filosa et al., 2006; Dunn and Nelson, 2010; Girouard et al., 2010). Mitochondria are enriched in PvAPs (Busija et al., 2016; Gӧbel et al., 2020), and nitric oxide (NO) synthesis from astrocytic mitochondria underlies vasodilation during mild hypoxia (Christie et al., 2023). Finally, astrocytes contribute to CBF regulation through CO_2_ sensing. In acute brain slices, local CO_2_ elevation induces a Ca^2+^ increase in astrocytes, leading to downstream COX1-dependent vasodilation (Howarth et al., 2017). Neurovascular coupling is not restricted to arterioles but also occurs at the capillary level mediated by pericytes contractility (Chaigneau et al., 2003; Peppiatt et al., 2006; Lacar et al., 2012; Hall et al., 2014; Hartmann et al., 2021). Indeed, preventing Ca^2+^ rise in astrocytes, by injecting a chelating agent or by inactivating IP_3_R2, inhibits capillary dilation, but has no effect on arteriolar dilation (Biesecker et al., 2016; Mishra et al., 2016). This regulation involves PGE2 produced by astrocytes acting on EP4 receptors on pericytes to induce capillary dilation (Mishra et al., 2016).

How this complex molecular machinery develops in astrocytes remains unknown. Due to technical limitations, many *ex vivo* studies have been conducted at mid-to-late postnatal stages in rodents, while *in vivo* studies have primarily focused on adult animals (Zonta et al., 2003; Mulligan and MacVicar, 2004; Mishra et al., 2016). Nevertheless, the ability of astrocytes to induce hemodynamic responses in BVs appears to emerge early during postnatal development.

### 5.4 Summary

Under physiological conditions, astrocytes play a key role not in the initial formation of the blood–brain barrier (BBB), but in its postnatal maturation and long-term maintenance. Several proteins expressed in PvAPs are involved in these regulatory functions. By forming a sheath around brain vessels and expressing water/ion channels as well as mechanosensors sensitive to shear stress, PvAPs structurally organize the flow and drainage of CSF and ISF. In addition, PvAPs are critical components of neurovascular coupling: they contribute to the postnatal maturation of arteriolar VSMCs and mediate the release of vasoactive molecules that regulate blood vessel tone.

### 5.5 Some open questions

- PvAPs progressively establish a physical barrier between BVs and the brain parenchyma during development. Could this regulates the distribution of signaling molecules between the vascular and the parenchymal compartments of the CNS?
- What are the signals from PvAPs that regulate postnatal maturation of VSMCs?
- It is currently unknown whether the increased contractility of VSMCs during postnatal development supports the maturation of the glymphatic system. When this last crucial physiological function first emerges during development has yet to be determined.
- The developmental regulation of neurovascular coupling by PvAPs needs to be more precisely defined, particularly in distinguishing between capillaries and maturing arterioles.

Neurovascular coupling underlies the blood oxygen level–dependent (BOLD) signal used in functional magnetic resonance imaging (fMRI), a technique increasingly employed in clinical and cognitive neuroscience. However, the BOLD signal differs between the developing and mature brain (Harris et al., 2011; Kozberg and Hillman, 2016).

- Whether astrocytes contribute to this change is an open question.

## 6 Diseases linked to early developmental alterations of PvAPs

**Figure 3:**
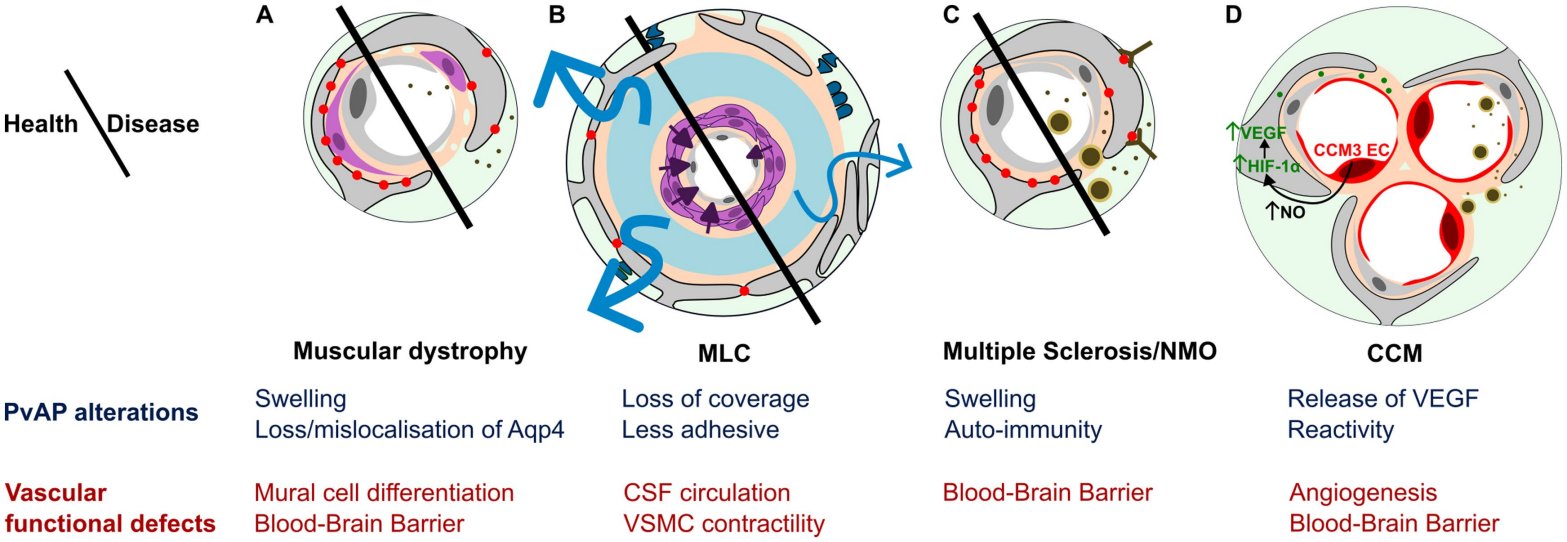
PvAP and GVU contribution to CNS diseases. **(A–D)** Schematic representation and summary of main PvAP alterations (blue) and GVU functional defects (red). **(A)** Muscular dystrophies (CMD and DMD): Swollen PvAPs and altered level/localization of Aqp4 is associated with patchy Basal Lamina, impaired mural cell differentiation and Blood–brain barrier leakage resulting in blood protein release in the brain parenchyma. **(B)** Megalencephalic leukoencephalopathy with subcortical cysts (MLC): Poorly adhesive PvAPs with reduced vascular coverage, presence of perivascular synapses/axons, mislocalized BL, altered CSF circulation, VSMC impaired contractile maturation, impaired arteriolar contractility and neurovascular coupling. **(C)** Autoimmune diseases: Multiple Sclerosis (MS) and neuromyelitis optica (NMO): Auto-immunity against PvAP proteins and swollen PvAP associated with leaky BBB with passage of blood proteins and peripheral immune cells into CNS parenchyma. **(D)** Cerebral cavernous malformations (CCM): Astrocyte could participate to CCM malformation via VEGF secretion driven by NO increase by CCM endothelial cells stabilizing HIF-1α in PvAPs. Astrocyte reactivity inducing BBB alteration and peripheral immune cells recruitment.

Neurogliovascular alterations are now considered as essential in many neuropathological contexts such as neurodegenerative diseases, inflammatory diseases, epilepsy as well as neurodevelopmental disorders (Ouellette and Lacoste, 2021). They include BBB and blood flow disruptions, inflammation and metabolic imbalance. Here, we will specifically focus on CNS disorders associated with potential defects in PvAPs, as well as vascular diseases that may be initiated by astrocyte-mediated perivascular dysfunction during development (Figure 3).

Brain vascular malformations. The two most common forms of vascular malformations of the brain are cerebral cavernous malformations (CCM) and arteriovenous malformations (AVM).

*CCMs* are low-flow lesions characterized by clusters of dilated capillaries and BBB dysfunction (Storkebaum et al., 2011). CCMs affect approximately 1 in 200 children and pose a lifelong risk of chronic and acute hemorrhage. Loss of function in three genes are responsible for CCM1 (*KRIT1*), *CCM2*, or CCM3 (*PDCD10*). CCM have mainly been described as endothelial cell-autonomous diseases, since endothelial-specific inactivation of the murine orthologous genes results in vascular lesions that closely resemble those observed in patients (Boulday et al., 2011; Chan et al., 2011). However, the mechanisms regulating initiation, progression and transition from quiescent to active CCM lesions remain to be determined, and might differ between CCM1, 2 and 3. Endothelial specific deletion of *Pcdc10* around birth leads to the postnatal formation of CCM lesions in the first 2 weeks (Lai et al., 2022), which is reduced by depletion of proliferative astrocytes in the hindbrain (Lopez-Ramirez et al., 2021). This suggests that proliferative astrocytes participate in the formation of vascular lesions. A mechanism implicating the increased production of nitric oxide (NO) by ECs was described (Figure 3). NO was shown to stabilize hypoxia-inducible factor 1α (HIF-1α) in astrocytes, inducing vascular endothelial growth factor (VEGF) expression by astrocytes, favoring angiogenesis and vascular lesions (Lopez-Ramirez et al., 2021). In a similar model, authors suggested an additional role for astrocytes in the transition to mature lesions: reactive astrocytes surrounding the lesions could participate to the recruitment of peripheral immune cells and the formation of the thrombus (Lai et al., 2022; Figure 3). *Pcdh10* deletion in neural cells could also lead to CCM (Louvi et al., 2011). Deletion in neural progenitors and astrocytes resulted in the apparition from 3 weeks of age, to lesions resembling human CCMs with thin-walled dilated clustered microvessels separated by single layers of endothelium (Louvi et al., 2011; Figure 3).

*AVM* are “fast-flow” lesions characterized by the formation of a “nidus,” where arterioles directly connect to veins (Storkebaum et al., 2011). Notably, the absence of PvAPs has been reported in perinidal capillaries (Tu et al., 2006). Furthermore, mutations in *Itgb8*, which encodes integrin *β*8, have been identified as a risk factor for AVM. Astrocyte-specific deletion of *Itgb8* in mice leads to an increase in vascular malformations in newly formed vessels, likely through impaired activation of TGF-β signaling (Su et al., 2010).

Leukodystrophies are monogenic disorders characterized by CNS white matter alterations (van der Knaap and Bugiani, 2017).

*Alexander Disease (AxD)* is caused by mutations in *GFAP* (Brenner et al., 2001). Three forms of the disease have been described, with child, juvenile, or adult onset. The most common form starts between birth and 2 years of age. It is characterized by seizures and spasticity and is generally lethal within 1 to 4 years (Li et al., 2005). The disease is characterized by aggregates termed *Rosenthal fibers* in PvAPs, which contain the protein chaperones αB-crystallin and HSP27 as well as GFAP (Sosunov et al., 2017). Mouse models with mutated forms of GFAP have been developed and reproduce early brain alterations, with the accumulation of Rosenthal fibers in PvAPs as early as 1 week after birth (Perng et al., 2006; Hagemann et al., 2009; Sosunov et al., 2017). GFAP is expressed not only in mature astrocytes but also in embryonic radial glia and adult neural stem cells, suggesting that GFAP mutations may affect the production and/or maturation of these progenitor populations as well. *In vitro* studies using AxD patient-derived induced pluripotent stem cells (iPSCs) and cortical organoids have shown reduced astrocyte production (Matusova et al., 2025). Additionally, a mouse model of AxD demonstrated impaired adult neurogenesis in the hippocampus, which was associated with cognitive deficits (Hagemann et al., 2013). Thus, while astrocyte dysfunction is considered the most prominent and well-documented contributor to disability in AxD, developmental defects affecting neural precursors may also play a role in disease pathogenesis.

*Megalencephalic Leukoencephalopathy with Subcortical Cysts (MLC)*. MLC is characterized by the presence of subcortical cysts visible on MRI (Leegwater et al., 2001; van der Knaap et al., 2012). Symptoms include macrocephaly, deterioration of motor functions with ataxia, spasticity, and eventually mental decline. The classic form is linked to recessive loss-of-function mutations in *MLC1* (around 75% of cases) or *HEPACAM* (around 20% of cases), which encode MLC1 and GlialCAM, two membrane proteins of the PvAPs (López-Hernández et al., 2011; Gilbert et al., 2019). Mouse models deleted for these two genes reproduce the human phenotype but without cysts (Hoegg-Beiler et al., 2014; Dubey et al., 2015; Bugiani et al., 2017; Gilbert et al., 2021). In *Mlc1* KO mice, an early developmental alteration of the GVU is observed including PvAPs defects (see Chapter 3), leading to low arteriolar contractility and impaired CSF drainage and neurovascular coupling (Figure 3; Gilbert et al., 2021; Slaoui et al., 2023). Recently, a screen for other proteins interacting with Mlc1 lead to the identification of new possible PvAPs actors of MLC (Alonso-Gardón et al., 2021), including Gprc5b and Gpr37l1, both expressed in PvAPs (Boulay et al., 2017; Passchier et al., 2023). Human mutations on *GPRC5B* and *AQP4* have also been found in MLC patients (Passchier et al., 2023).

*Leukodystrophy linked to CLC-2.* CLC-2 is a chloride channel encoded by the gene CLCN2 and expressed in the plasma membrane of almost all mammalian cells, including astrocytes (Blanz et al., 2007; Depienne et al., 2013). Loss of function mutations in *CLCN2*, initially thought to be implicated in MLC, are responsible for another form of leukodystrophy with a delay onset between 1 and 12 years (Depienne et al., 2013). They lead to retinal and testicular degeneration and leukodystrophy, whereas gain-of-function mutations cause hyperaldosteronism (Depienne et al., 2013). CLC-2 is critical for extracellular ion homeostasis (Göppner et al., 2021). In human white matter, CLC-2 forms a complex with GlialCAM and MLC1 in PvAPs (Jeworutzki et al., 2012; Depienne et al., 2013; Sirisi et al., 2017). Inactivation of *Clcn2* in the mouse leads to progressive vacuolization of the white matter in the brain and spinal cord, starting before P28, and is associated with progressive leakage of the BBB (Blanz et al., 2007). Comparison of *Mlc1, Hepacam*, or *Clcn2* deleted mouse models has helped to clarify the mechanisms underlying leukodystrophies (Hoegg-Beiler et al., 2014; Göppner et al., 2021). Mlc1 and GlialCAM are required for Clc-2 localization. However, Clc-2 is dispensable for the GlialCAM/Mlc1 complex formation at the membrane, which could also explain the distinct pathophysiologies related to these genes (Hoegg-Beiler et al., 2014), CLC-2 mutations primarily disrupting ionic balance and cellular excitability, while GlialCAM/MLC1 mutations interfering specifically with glial membrane interactions.

Autoimmune disorders form a group of neurological diseases driven by autoimmunity against CNS proteins. Two diseases in particular have been related to PvAPs, *Neuromyelitis optica* (NMO) and *Multiple Sclerosis* (MS). They generally start in the young adult. MS is characterized by the apparition of focal lesions of inflammation and demyelination around postcapillary venules or in the periventricular region. Most NMO patients are positive for anti-Aqp4 antibodies (Segal et al., 2025). Interestingly, these antibodies require the presence of the read-through isoforms Aqp4ex to recognize the Aqp4 OAPs in PvAPs (Palazzo et al., 2019). This binding induces complement activation and astrocyte death (Segal et al., 2025). In acute MS lesions, swelling and damage of PvAPs are early events (Figure 3). B-lymphocyte related autoimmunity against PvAP proteins have also been described, including Kir4.1, GlialCAM and MLC1 (Srivastava et al., 2012; Lanz et al., 2022; Dahl et al., 2025). Alteration of PvAPs through autoimmune targeting of their associated proteins, particularly in the context of neuroinflammation, could represent a triggering event in this group of neurological diseases (van der Knaap and Min, 2025).

Muscular dystrophies (MD) are a group of disease of genetic origins. They have in common to affect peripheral muscles with CNS comorbidities (Figure 3).

*Congenital muscular dystrophy* (CMD) is a progressive dystrophy caused by mutations in *LAMA2* encoding the laminin α2 chain which is necessary for laminin 211 formation (see chapter 4). Its absence results mainly in skeletal muscle damage: patients with null-expression mutations have strong muscle alteration from birth and often die of muscle wasting and respiratory failure before adult age (Yurchenco et al., 2018). In addition to muscular dystrophies, patients show accompanying brain abnormalities, including seizures, perturbed cortical development and MRI white matter changes, indicative of alteration in myelination possibly due to BBB defects (Caro et al., 1999). *Lama2* is expressed by both astrocytes and pericytes and serves as a key component of the BL. Consistent with patient outcomes, *Lama2* knockout mice exhibit a markedly shortened lifespan, dying before 4 weeks of age (Menezes et al., 2014). In the brain, deletion of laminin α2 or astrocyte-derived laminin γ1 disrupts the formation of the GVU, leading to mislocalization of AQP4 in PvAPs and impaired pericyte differentiation. Moreover, the absence of astrocytic laminin γ1 compromises BBB integrity (Figure 3; Menezes et al., 2014; Yao et al., 2014).

*Duchenne muscular dystrophy* (DMD) is a X-linked disease caused by mutations in *DMD* encoding dystrophin. Absence of the protein leads to muscle degradation and ultimately to cardiac and respiratory failure. The CNS is also affected, with attention deficit, hyperactivity, obsessive compulsive disorder, anxiety and sleep disorders (Emery, 2002). Approximately 8% of patients experience epilepsy (Hendriksen et al., 2018). Several isoforms of DMD are produced by alternative splicing and promoters. The main brain dystrophins are Dp427, Dp140, and Dp71 (numbers refer to the molecular weight). They all bind the DAPC, which links the BL to intracellular actin cytoskeleton and signaling proteins in PvAPs. Loss of Dp71 is considered the primary contributor to CNS-related symptoms (Daoud et al., 2009). In addition to alteration of synapse identity and structure (Chaussenot et al., 2019), Dp71 deletion in the mouse alters Aqp4, *β*-DG and Kir4.1 levels in PvAPs (Belmaati Cherkaoui et al., 2021). Mdx mice, with a point mutation in the DMD gene, also display altered Aqp4 localization in PvAPs associated with impaired BBB integrity (Frigeri et al., 2001; Nico et al., 2004).

### 6.1 Summary

All of these diseases share a common feature: disruption of gliovascular functions—either as a primary cause or a secondary consequence. These alterations can be broadly categorized as follows:

- Neuroinflammation and astrocyte reactivity in autoimmune diseases such as MS and NMO, as well as cerebral CCMs.
- Water/ionic flux and glymphatic CSF altered circulation in leukodystrophies caused by the loss of MLC1, GlialCAM or CLC-2. More recently, ionic imbalance has also been proposed as a phenotype of MS.
- Cerebral blood flow alteration in MLC and vascular malformations linked to AVM and CCM.
- Angiogenesis alteration in vascular malformations (AVM, CCM).
- BBB breakdown in muscular dystrophies (e.g., CMD, DMD), autoimmune disorders (MS, NMO), and vascular malformations (AVM, CCM).

These diseases can also be classified based on the involvement of PvAPs:

- Genetic diseases with primary defects in PvAP proteins in leukodystrophies (mutations in *MLC1*, *GlialCAM*, *CLC-2*) and muscular dystrophies (mutations affecting Dp71 and laminin α2). In animal models, these mutations have been shown to alter PvAP architecture and impair GVU functions, supporting a causal role in disease pathogenesis.
- Autoimmune diseases where PvAP proteins are targeted in MS and NMO.
- Secondary PvAP alterations in AVM (disrupted PvAP coverage), and in CMD and DMD (mislocalization of AQP4).

Overall, these conditions can be described as disorders of the late neurodevelopment. Most show delayed onset and are progressive.

### 6.2 Some open questions

- Whether defects in PvAPs are the initiating event in disease pathogenesis remains largely unknown—even in genetic disorders directly affecting PvAP-associated proteins. The most documented example is the MLC mouse model, where GVU abnormalities are observed prior to the appearance of white matter defects. This early GVU disruption may serve as a triggering event by impairing VSMC contractility in the early postnatal period. The resulting dysfunction in neurovascular coupling could lead to metabolic imbalance, potentially contributing to the formation of intramyelinic edema (Gilbert et al., 2021). Indeed, myelination is an energy-intensive process, and growing evidence suggests that impaired brain energetics plays a key role in the onset and progression of leukodystrophy (Cunnane et al., 2020).
- Long term consequences of early alterations of the GVU need to be addressed. In particular, modification in homeostatic balance by altered function of ionic and water channels, causing impaired CSF circulation, could make the brain more susceptible to pathologies (Ouellette et al., 2020), by accumulation of toxic molecules (Rasmussen et al., 2018). Defects in glymphatic development could be implicated in diseases such as neonatal hydrocephalus (Emmert et al., 2019). They could have long term consequences on brain functions (Song et al., 2024).

## 7 Concluding remarks

PvAPs play a crucial role in maintaining the intricate interface between the brain’s neural and vascular systems, collectively forming the GVU. Their diverse functions—ranging from regulating the BBB permeability, ensuring neurovascular coupling, and maintaining extracellular homeostasis—are vital for proper brain function and metabolic balance. As our understanding of PvAPs continues to evolve, it becomes evident that these processes are highly heterogeneous, with distinct mechanisms regulating their formation and function depending on vessel type, astrocyte subtype, and developmental stage. Despite the progress in our comprehension of the cellular and molecular underpinnings of PvAPs, significant gaps remain. The mechanisms guiding their formation, the role of local translation in their development, and the precise molecular pathways they use to interact with vascular cells are still poorly understood. Studies on the perisynaptic organization of PvAPs and their contribution to the diversity of neurovascular functions remain sparse. Possible interplay between PvAPs and neural cells migrating on BVs as well as with neurons during development is yet unknown. A major challenge in current research lies in the difficulty of studying PvAPs from contiguous astrocytes. Additionally, the role of astrocytes in postnatal angiogenesis and the maturation of the BBB highlights their involvement in long-term brain vascular integrity. More research is needed to explore the involvement of PvAPs in these processes. Importantly, the plasticity of PvAPs, i.e., their ability to reform and adapt in pathological situations, suggests an additional layer of regulation that could offer novel therapeutic avenues. As we continue to explore the developmental mechanisms, molecular pathways, and broader implications of PvAPs in health and disease, it will be critical to refine experimental models and methods, such as proteomics and advanced imaging techniques, to address these unanswered questions.

In conclusion, while the field has made significant strides in understanding the structure and function of PvAPs, future studies must focus on the unexplored dimensions of their development, organization, molecular repertoire, and roles in brain pathology. By investigating how early developmental alterations in PvAPs contribute to neurovascular dysfunction, we may uncover new insights into brain diseases that could lead to more effective treatments and preventive strategies.
